# When, where, and why should we look for vestibular dysfunction in people with diabetes mellitus?

**DOI:** 10.3389/fresc.2023.1306010

**Published:** 2024-01-11

**Authors:** Frank E. DiLiberto, Heather E. R. Kamath, Maxine L. Olson, Marcello Cherchi, Janet O. Helminski, Michael C. Schubert

**Affiliations:** ^1^ Department of Audiology and Speech Pathology, Captain James A. Lovell Federal Health Care Center, North Chicago, IL, United States; ^2^ Department of Physical Therapy, Rosalind Franklin University of Medicine and Science, North Chicago, IL, United States; ^3^ Neurology, University of Chicago Medicine, Chicago, IL, United States; ^4^ Laboratory of Vestibular NeuroAdaptation, Department of Otolaryngology-Head and Neck Surgery, Johns Hopkins University, Baltimore, MD, United States

**Keywords:** vestibular, diabetes mellitus, utricle, saccule, semicircular canal, balance

## Abstract

The biochemistry of diabetes mellitus results in multi-system tissue compromise that reduces functional mobility and interferes with disease management. Sensory system compromise, such as peripheral neuropathy and retinopathy, are specific examples of tissue compromise detrimental to functional mobility. There is lack of clarity regarding if, when, and where parallel changes in the peripheral vestibular system, an additional essential sensory system for functional mobility, occur as a result of diabetes. Given the systemic nature of diabetes and the plasticity of the vestibular system, there is even less clarity regarding if potential vestibular system changes impact functional mobility in a meaningful fashion. This commentary will provide insight as to when we should employ diagnostic vestibular function tests in people with diabetes, where in the periphery we should look, and why testing may or may not matter. The commentary concludes with recommendations for future research and clinical care.

## 1 Introduction

Diabetes mellitus (DM) is a worldwide health concern. Approximately 800 billion in annual medical costs are attributed to the care of over 400 million people with DM worldwide (1). For these individuals, abnormal glycemic control propagates a cascade of biochemical processes that lead to multi-system tissue compromise. In compounding fashion, cardiovascular, renal, orthopaedic, and sensory changes combine to reduce health, quality of life, and the level of functional mobility and physical activity needed to regulate blood glucose and mitigate further tissue compromise (1–5).

**Figure 1:**
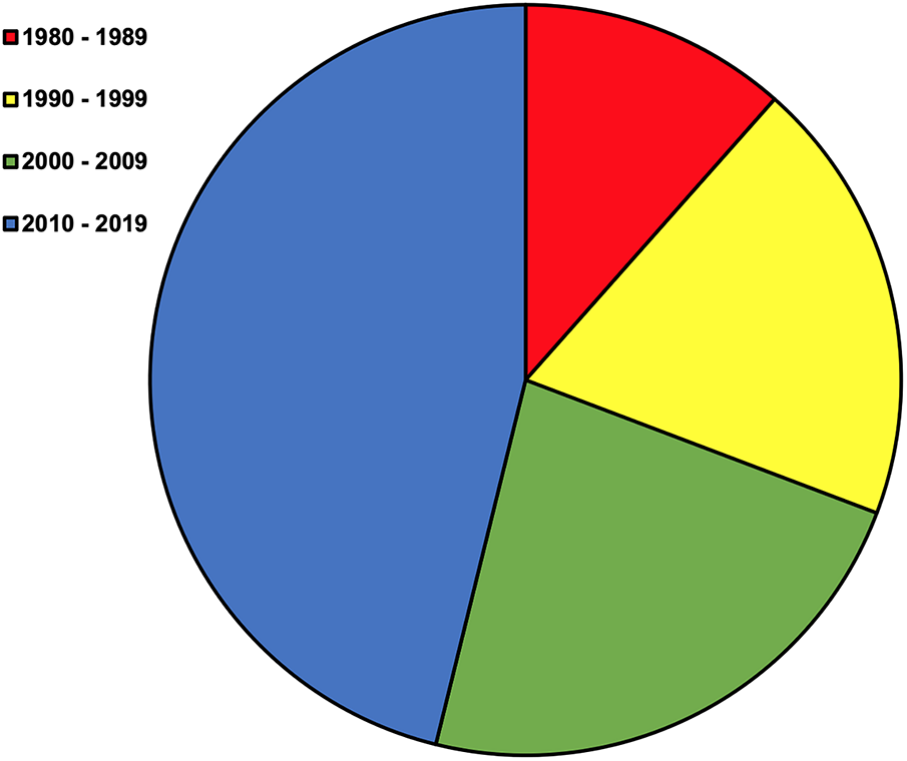
Original manuscripts by decade beginning in 1980. The term diabetes mellitus was combined with a search for vestibular OR inner ear in PubMed on July 24th 2023. This resulted in 420 articles. Review of titles, abstracts, articles, and subsequent snowball sampling was performed to identify 26 original research manuscripts written in English on peripheral vestibular diagnostic testing or structure in people or animals with DM without vestibular attributed symptoms of dizziness.

Well-characterized sensory system pathology, such as peripheral neuropathy and retinopathy, are particularly prevalent and detrimental to functional mobility in people with DM (2). More specifically, the prevalence of peripheral neuropathy and/or retinopathy may be higher than 70% and is associated with reduced balance and elevated fall risk (2, 3, 6–10). With this impetus and coalescent research, indications and methods for screening peripheral neuropathy and retinopathy have been developed and translated into standard clinical practice (11). However, research exploring if the same biochemistry propagating peripheral neuropathy and retinopathy also affects the third essential sensory system for balance and mobility, the vestibular system, is much less cohesive. Lack of cohesion in the field has precluded clinical recommendations for vestibular screening and rehabilitation approaches, and persists despite an increasingly concerted effort (Figure 1). Nevertheless, interest in this topic continues given histopathologic evidence, the importance of vestibular system signaling and rectification of altered sensory inputs (e.g., somatosensation), the responsiveness of the vestibular system to non-invasive interventions, and irrespective of inherent challenges to vestibular assessment (12–14).

Assessment of vestibular function in the context of DM is arguably more challenging than the evaluation of other sensory systems. While patients are able to readily report pain, paresthesia, and numbness or visual changes indicating peripheral neuropathy of the feet and retinopathy, the symptoms of vestibular dysfunction may not be as readily perceived. DM affects organs and tissues that depend on microvasculature blood supply and additional biochemical reactions, in a bilateral and relatively symmetrical fashion (e.g., feet, eyes, kidneys) (11, 15). This expected pattern of insult within the inner ear that depends on a similar type of local homeostasis, introduces the likelihood of vestibular signaling decline without the degree of asymmetry typically seen in symptomatic (e.g., dizzy) patients. Further, the ability of the vestibular nuclei and cerebellum to reintegrate altered vestibular signals for appropriate motor responses introduces the possibility of central compensation (16). This plasticity presents further challenges with respect to the timing of assessment, determining a meaningful degree of loss, and predicting the likelihood of functional consequences. In this way, it is quite possible that vestibular dysfunction is present, asymptomatic, and even functionally inconsequential to a varying degree in people with DM. We suggest appreciation of this level of complexity is needed to glean insight into current research examining how hyperglycemia affects vestibular function.

The intent of this commentary is to review the state-of-research on peripheral vestibular function in people with DM without diagnosed vestibular pathology or symptoms of dizziness. A mini-summary of the peripheral vestibular system and diagnostic tests (Tables 1, 2), and review of DM pathophysiology and histopathological studies are used to enrich our interpretation and readers' perspective on human studies. Commentary is then organized to identify the most likely indicators and location of dysfunction, and discuss why continued research is needed to substantiate the need for vestibular testing in the absence of patient symptoms.

**Table 1:** The peripheral vestibular system.

Peripheral structure	Anatomy	Physiological functions
Otolith • Saccule • Utricle	Within a sac-like structure (otolith) are saddle shaped structures of sensory epithelium (maculae) comprised of kinocilia and stereocilia hair cells. The hair cells project into a gelatinous layer (otolithic membrane). The otolithic membrane is weighted with embedded otoconia (calcium carbonate crystals). • Saccular maculae oriented vertical • Utricular maculae oriented horizontal	Linear acceleration (e.g., head tilts and translational movements) causes movement of the weighted membrane and deflection of the hair cells generating either excitation or inhibition of the vestibular afferent. The saccule (vertical and anterior/posterior translations) and utricle (horizontal anterior/posterior and lateral) transmit signals via the inferior (from sacculus) and superior portion (from utricle) of the vestibular nerve to the vestibular nuclear complex. The vestibular nuclear complex generates a postural response (VSR) and/or eye movement (ocular tilt reaction and translational VOR).
Semicircular Canals • Anterior • Posterior • Horizontal	Endolymph-filled canals of one side are oriented orthogonally to each other. The two sides are arranged to work together as co-planar pairs (e.g., right anterior and left posterior (RALP), left anterior and right posterior (LARP), and horizontal canals). Each canal has an enlarged area called the ampulla. It contains an area of sensory epithelium consisting of hair cells (kinocilia and stereocilia) that project into the membranous diaphragm, the cupula.	Angular acceleration causes deflection of the hair cells. The orientation of the hair cells and arrangement of the co-planar canal pairs will determine if rotation in the plane of the canal will deflect the hair cells and cause excitation or inhibition of the afferent from one of the canal pairs. The inferior branch of the vestibular nerve originates from the posterior canal. The superior branch originates from the anterior and horizontal canal. Information from the vestibular nuclear complex is used to generate angular VOR and VCR.
Vestibulocochlear Nerve—CN VIII	The superior and inferior divisions of the vestibular branch of CN VIII innervates the five end-organ structures. Primary vestibular afferents form three types of endings around hair cells. Calyceal endings on Type I hair cells, bouton endings on Type II, and dimorphic endings both Type I and II hair cells.	The hair cells convert otolith and canal mechanical stimuli to neural action potentials and increase or decrease the tonic resting firing rate of CN VIII. The firing rate of the vestibular afferent may be classified based on its discharge regularity, either regular or irregular. The vestibular afferents synapse in the vestibular nuclear complex (superior, inferior, medial, and lateral nuclei) and cerebellum where the information is processed with other sensory input and a vestibular motor response is determined.

CNS, central nervous system; CN, cranial nerve; VOR, vestibulo-ocular reflex; VSR, vestibulospinal reflex; VCR, vestibulocollic reflex; SCC, semicircular canal.

The peripheral vestibular system consists of vestibular receptors and afferents. The vestibular receptors are hair cells within the otolith that detect linear acceleration and cristae ampullaris of the ampulla of each of the three semicircular canals that detect angular rotation. The afferent is the superior and inferior branch of the vestibular nerve. The primary afferents project to the central nervous system, the vestibular nuclear complex located dorsolateral to the junction of the pons, medulla, and the vestibulocerebellum. The vestibular nuclear complex processes vestibular, proprioception, and visual information. The vestibular nuclear complex projects to the three ocular motor nuclei for gaze stability (VOR), to the spinal cord for postural control such as protective extension (VSR) and head righting (VCR), and to the cortex via the thalamus for perception and spatial navigation.

**Table 2:** Peripheral vestibular system tests (17–19).

Peripheral organs	Tests
Utricle	oVEMP: An auditory stimulus is delivered, and inferior oblique muscle activity is recorded with surface EMG as the patient sits with a 30° upward gaze (typical position). An absent response, low amplitude response, or longer latency to onset of the extraocular muscle response indicates abnormality of the utricular pathway.
	SVV: In sitting, patients are asked to indicate when a slowly rotating line, projected in front of them, is in the vertical position. Error is the difference in patients’ perception vs. actual vertical. Static tests with greater than 2° of error are considered abnormal. Dynamic tests that manipulate the visual system (optokinetic backgrounds) are considered abnormal when error increases from the static value. Dynamic tests while the patient turns in an offset (off axis) rotational chair, stimulates one utricle at a time, and is abnormal when error decreases from static error.
Saccule	cVEMP: A auditory stimulus is delivered, and sternocleidomastoid muscle activity is recorded with surface EMG as the patient lays supine with an active rotation and head lift of 30° (typical position). An absent or low amplitude response, or longer latency to onset of the sternocleidomastoid muscle response indicates abnormality of the saccular pathway.
Semicircular Canals	Calorics: Warm and cool water (or air) is delivered to the external auditory canal of each ear individually as the patient lays with the head elevated 30° from supine. The temperature gradient serves as a low-frequency (.003 Hz) stimulus and induces nystagmus. Oculography is used to record slow phase eye velocity. Velocity, duration of the response, symmetry, and directional preponderance are used to evaluate the activated horizontal SCC function. An asymmetry of >25% is a common metric indicative of a unilateral weakness.
	Rotational Chair: The patient undergoes passive sinusoidal harmonic acceleration at low-to-mid-range frequencies (typically 0.01–0.64 Hz) while seated in the chair. Oculography is used to record slow phase eye velocity of nystagmus. VOR gain (eye velocity/head velocity) and phase (head vs. eye position in degrees) are the main outcomes of this bilateral horizontal SCC test. Values ±2 SD of laboratory norms at a given frequency are considered abnormal.
	Active or Passive Head Thrust Tests: In sitting, head neutral, patients gaze upon a target at a set distance. A brisk (>150 °/s), low amplitude (10°), head rotation in the plane of the canal either actively induced by the patient, or passively induced by a clinician/researcher, serves to excite a SCC in the direction of the head thrust (e.g., right anterior and left posterior; right posterior and left anterior; and right and left horizontal canals). Abnormal VOR gain and/or saccadic eye movements to maintain/restabilize gaze on the target are typically used to indicate abnormal function of the stimulated SCC. Dynamic visual acuity (DVA) and video head impulse testing (vHIT) are examples of this high frequency (≈1–6 Hz) testing paradigm.

oVEMP, ocular vestibular evoked myogenic potential; EMG, electromyography; SVV, subjective visual vertigo; cVEMP, cervical vestibular evoked myogenic potential; DVA, dynamic visual acuity; vHIT, video head impulse test; SCC, semicircular canal.

Brief summaries of test procedures, outcomes, and abnormal findings are provided.

## 2 Pathophysiology

Hyperglycemia defines DM. Laboratory tests are used to measure blood glucose levels and diagnose DM (e.g., HbA1c ≥ 6.5%). Type 1 and Type 2 are the most common classifications of DM in the population (20). Inability of pancreatic cells to produce insulin is the primary etiology for the typically earlier onset Type 1, or insulin dependent DM (IDDM). Inability of receptor cells to receive and utilize circulating insulin is the primary etiology of Type 2, or non-insulin dependent DM (NIDDM). Type 2 DM is most prevalent and associated with lower physical activity and higher body mass index (BMI) or weight. However, overlap between these DM classifications is recognized and ongoing research may reveal more precise classifications (20). Until then, heterogeneity of patient presentations, including the type, severity, and sequencing of cellular level tissue damage will remain a challenging reality of medical care.

Certain cells, such as endothelial cells, are ill equipped to reduce the transport of glucose across its membrane when confronted with elevated blood glucose (21). Inability to regulate the influx of glucose at the cellular level breeds excessive reactive oxygen species and leads to oxidative stress. Oxidative stress initiates a cascade of reactions via multiple pathways that result in tissue damage (21, 22). While each pathway deserves consideration, the contribution of excessive advanced glycation end products (AGEs) to cellular functions is often attributed to DM related tissue damage (retinopathy, nephropathy, peripheral neuropathy), and theorized to also cause peripheral vestibular dysfunction (13, 21, 23). Among numerous effects, intracellular, extracellular, and circulation of AGE precursors leads to observable changes in extracellular matrix, collagen and neural tissues, and small vessel characteristics which reflect microangiopathy (e.g., increased basement membrane and wall thickness). Importantly, the combination of altered tissue structure and reduced diffusion of nutrients to tissues serves to both increase and accelerate dysfunction. Animal model and post mortem human studies present the opportunity to evaluate if these mechanisms of tissue damage manifest in the peripheral vestibular system.

## 3 Histopathology studies

While early histopathological work did not detect small blood vessel changes supplying the peripheral vestibular system (24), subsequent animal model research has detected morphological alterations within the vestibule (Table 3). Across a series of light and electron microscopy case-control studies Meyers and colleagues demonstrated peripheral vestibular system changes in medically induced DM (i.e., Type 1) animal models (25–28). Characteristic signs of microangiopathy were not observed. However, the observation of DM group capillary proliferation in the otolith, in one of two studies, was theorized to be a compensatory adaptation to metabolic stress (poor oxygen diffusion). Interestingly, renal microangiopathy was observed in the group with otolith capillary proliferation; suggesting the vestibule potentially adapts differently to metabolic stress. Additional unique otolith alterations, reflecting metabolic stress and in part characteristic of AGEs, include the excessive extracellular matrix and connective tissue lipid droplets within the otolith that were correlated or trended with higher blood glucose levels. A variable degree of adaptations in myelin structure, including greater lysosomal activity with higher levels of blood glucose, were also observed in nerves supplying the otolith. Lastly, typical neuropathic changes, such as axonal dwindling and myelin sheath thinning, were observed in nerves to the horizontal semicircular canals and in relation to longer DM duration; but not in otolith nerves, along CN VIII (unpublished data), or in relation to blood glucose levels. In total, these morphological changes would be anticipated to reduce the quality and rate of end organ signals in people with DM.

**Table 3:** Histopathological studies.

	Sample	Main findings (DM compared to controls)
Meyers et al. (25)	• Sprague-Dawley rats • 28 DM (Streptozotocin) • 19 controls	• No basil lamina thickening of otolith • Larger CSA of utricle (18.5%) and saccule (26%) attributed to increased capillary diameter and density
Meyers et al. (26)	• Sprague-Dawley rats • 27 DM (Streptozotocin) • 14 controls	• Greater secondary lysosomes and lipid droplets in connective tissue cells, and excessive extracellular matrix, of utricle and saccule maculae • Saccule Type I hair cell degeneration in a small subset (*n* = 2) of the longer duration DM animals
Meyers (27)	• Sprague-Dawley rats • 10 DM (Streptozotocin) • 8 controls	• Saccule and utricle nerve myelin sheath changes included disrupted lamellae, lysosomal bodies, peri-axial expansion of Schwann cell bodies. • Lysosomal digestion of portions of myelinated nerve fibers in a subset • No microangiopathy signs or demyelination/axonal degeneration observed
Meyers et al. (28)	• Sprague-Dawley rats • 16 DM (Streptozotocin) • 9 controls	• Thinner myelin sheaths of hSCC nerves; thinnest with longer duration DM • Smaller nerve fibers with larger intrasheath diameters • Larger number of nerve fibers; greatest with higher blood glucose
Perez et al. (29)	• Sand rats • 7 DM (diet induced) • 7 controls	• Longer latency and lower amplitude vestibular evoked potential utricular responses from linear translations
Kocdor et al. (30)	• Post mortem humans • 39 DM (16 T1, 23 T2) • 40 age matched controls	• No difference in saccule arteriole wall thickness or Type II hair cell density • 16%–17% lower Type I hair cell density

CSA, cross sectional area; hSCC, horizontal semicircular canal; T1, Type 1 DM; T2, Type 2 DM.

Summaries of six studies examining the constitution and function of the peripheral vestibular system are presented.

More recent work further supports the likelihood of otolith dysfunction due to DM (Table 3). Perez et al. (29) demonstrated utricular pathway dysfunction in a diet induced (i.e., Type 2) DM animal model. Whether this finding was isolated to the vestibule or changes at longer durations of DM is unclear. Additionally, Kocdor et al. (30) identified loss of saccule Type I hair cells (calyceal endings) in people with Type 1 and 2 DM compared to controls, but did not find evidence of saccule microangiopathy. Combined, both studies point to a vulnerability of otolith Type I hair cells to hyperglycemia.

The above histopathologic research can frame expectations and interpretation for human subject studies evaluating peripheral vestibular function in people with DM. First, and in general, DM can create structural changes in the saccule and utricle, as well as in the nerves supplying the otolith and horizontal semicircular canals. Unique otolith capillary responses, without evidence of overt microangiopathy, as well as the collagenous/extracellular matrix and neural responses appear to be related to metabolic stress/AGEs in relation to elevated blood glucose. More characteristic signs of neuropathy may only be present in nerves supplying the semicircular canals, and in relation to longer duration DM. Further, while the anatomy (i.e., collagen type, short length of CN 8) (25) of the inner ear may underlie its unique response, at least short term, vestibular dysfunction may occur regardless of other microangiopathy signs (e.g., retinopathy, nephropathy) and potentially along with signs of AGE effects of the feet (e.g., peripheral neuropathy) (23, 31). Accordingly, key predictive factors of consideration in human studies include blood glucose level, duration of DM, and the presence of peripheral neuropathy (PN). Additionally, given the limited scope of the above research (i.e., semicircular canal not assessed in diet induced DM), known age related changes and possible sex differences of the vestibular system, and relationship of obesity to elevated blood glucose, we suggest DM Type, age, sex, and BMI are also worth consideration (20, 32, 33). Lastly, the anticipated location of vestibular dysfunction includes the entirety of the vestibule, with a possibility that tests biasing Type I hair cell function (i.e., high frequency tests; Table 2) may prove more sensitive in the detection of vestibular dysfunction in people with DM.

## 4 Human studies

In 1961, Jorgensen and Buch provided a historical record of publications on vestibular function in people with DM (34). While details are limited, vestibular dysfunction in people with DM was detected prior to 1915. Two authors found evidence of dysfunction in a small amount of people with DM reporting dizziness, whereas an additional larger study did not detect abnormal rotatory or caloric function tests in people with DM. In the 1960′s two additional studies demonstrated horizontal semicircular canal dysfunction via caloric testing; which were often bilateral, as opposed to unilateral, in presentation. However, a third study only found caloric abnormalities in 2/69 people with DM (34). It is challenging to draw conclusions from this early time period of investigations. However, these works recognized the potential for DM to impair inner ear function and set the stage for future research as DM prevalence and life expectancy increased, and as vestibular diagnostic testing advanced.

### 4.1 When do we screen for peripheral vestibular dysfunction?

Patient and disease specific factors may contribute to vestibular dysfunction in people with DM. In recognition of this possibility, sample characteristics such as DM classification, participant age, and sex have been consistently reported in manuscripts examining vestibular function in people with DM without dizziness (Table 4). However, BMI and disease severity factors (e.g., HbA1c, DM duration, PN), while rooted in established DM pathophysiology, are inconsistently reported. Disease specific factors appear to be particularly important considerations given those with DM and higher HbA1c and/or longer disease duration perform worse on balance tests which exploit vestibular integration (standing on foam with eyes closed) (58). Herein we comment on each factor to provide clues as to which patients may be more likely to have peripheral vestibular dysfunction.

**Table 4:** Human subject vestibular diagnostic testing studies.

Article	Sample characteristics	Findings
1st author (year)	• *n* Group; % Female; Age; BMI • HbA1c; DM Duration; *n* PN	**Vestibular end-organ pathway:** result
Aantaa (1981) (35)	• 24 DM T1; 50%; 34 years old • NR; 12 years; 9 PN	**hSCC:** ≥6 (25% of sample) with abnormal caloric tests
Biurrun (1991) (36)	• 46 DM T1; 8%; 26 years old • 9.4%; 9 years; 16 PN • 3 HC; NR; 26 years old	**hSCC:** 22 (48% of sample) with abnormal caloric tests
Chamyal (1997) (37)	• 30 DM T1 (10) T2 (20); 88%; <50 years old • 30 HC; 50%; <50 years old	**hSCC:** Normal caloric testing
Sharma (1999) (38)	• 25 DM; 48%; ≤50 • 25 DM w/comp.; 48%; ≤50 years old • 5 HC; 48%; ≤50	**hSCC:** Normal caloric testing
Gawron (2002) (39)	• 95 DM T1; 51%; 16 years old • 44 HC; 55%; 16 years old	**hSCC:** Increase slow phase eye velocity (1.5–4 deg/s) and 11 (12% of sample) with abnormal caloric testing compared to HC (2 abnormal tests)
Nicholson (2002) (40)	• 41 DM T1 (18) T2 (23); 39%; ≈63 years old • 45 HC; 60%; 61 years old	**hSCC:** VOR phase but not gain group differences in active or passive horizontal head rotation testing.
Klagenberg (2007) (41)	• 30 DM T1; 43%; 26 years old	**hSCC:** 18 (60% of sample) with abnormal caloric tests
Rigon (2007) (42)	• 19 DM T1; 53%; ≤25 years old • 19 HC; 53%;	**hSCC:** 18 (36% of DM sample) with abnormal responses and DM group with significantly lower caloric responses than HC.
Bektas (2008) (43)	• 38 DM T2; 50%; ≈51 years old • NR; ≈7 years; 25 PN • 21 HC; 43%; 49 years old	**Saccule:** No group differences in cVEMP latencies or inter-amplitude regardless of PN status.
Kamali (2013) (44)	• 24 DM T1; 42%; ≤40 years old • NR; NR; 10 PN • 24 HC; 54%; ≤40 years old	**Saccule:** Group differences (DMPN, DM, HC) in cVEMP latencies explained by longer latencies in DMPN vs. HC. No between group inter-amplitude differences.
Konukseven (2015) (45)	• 30 DM T2; 53%; 44 years old • 9.1%; 5 years; excluded • 30 Pre-DM; 50%; 46 years old • 5.7%; NA • 31 HC; 51%; 45 years old • 5.0%; NA	**Utricle:** 10 (34% of DM sample) with abnormal oVEMP. No between group difference in inter-amplitudes, but significantly longer latencies in DM group. **Saccule:** 17 (57% of DM sample) with abnormal cVEMP. No between group difference in inter-amplitudes, but significantly longer latencies in DM group.
Razzak (2015) (46)	• 47 DM T2; 28%; 57 years old; 30 kg/m^2^ • 7.1%; 10 years; excluded • 29 HC; 31%; 57 years old; 27 kg/m^2^	**Utricle:** No between group difference in static SVV conditions. Both groups had greater error with dynamic condition (tilted frame). DM group had significantly greater error than HC group in dynamic condition.
Sahu (2015) (47)	• 15 DM T2; 50 years old • 15 HC; 52 years old	**Saccule:** 16/30 DM group ears (vs. 0 HC) with absent cVEMP responses. Significantly lower DM group inter-amplitudes, but no group difference in latencies.
Ward (2015) (48)	• 25 DM T2; 40%; 65 years old; 32 kg/m^2^ • 8.3%; 18 years; 3.5 MNSI • 25 HC; 52%; 64 years old	**Utricle:** 46% absent or delayed n1 with oVEMP testing in DM group (vs. 12% in HC). Significantly lower n1 amplitude in DM group. **Saccule:** 32% absent cVEMP test in DM group (vs. 12% HC). Significantly lower inter-amplitude cVEMP in DM group. **SCC:** 70% of DM group had at least one abnormal canal with passive DVA (disappearing “E”) test. DM group with significantly worse hSCC and aSCC DVA than HC.
Jauregui-Renaud (2017) (49)	• 101 DM T2; 73%; 60 years old; 29 kg/m^2^ • 7%; ≈8 years; ≈30 PN • 51 HC; 57%; 57 years old; 28 kg/m^2^	**Utricle:** Significantly worse error with static SVV in DM group, though error was <2°. Significantly more error in HC group vs. DM group in dynamic SVV during off-axis rotation. **hSCC:** No between group difference in VOR gain at.16 and 1.28 Hz rotational chair testing
Kalkan (2018) (50)	• 66 DM T2; 56%; 54 years old • NR; 7 years; 33 w/o PN • NR; 11 years; DMPN; 33 PN • 35 HC; 45%; 50 years old	**Utricle and Saccule:** Significantly lower inter-amplitude with oVEMP and cVEMP testing in DM groups vs. HC, but no difference between DM w/o PN and DMPN groups. No group differences in latencies. **SCC:** No between group difference in median VOR gain with vHIT testing
Kanumuri (2018) (51)	• 40 DM T2; 30%; <60 years old • NR; >5 years; NR • 20 HC; NR	**Saccule:** 4 (25% of asymptomatic DM subgroup) with longer cVEMP latencies
Omar (2018) (52)	• 8 DM T2; NR; 37 years old • NR; <5 years; 0 • 8 HC; NR; 35 years old	**Utricle and Saccule:** No between group differences in inter-amplitudes or latencies of cVEMP or oVEMP testing. Trend of worse DM group inter-amplitudes was noted. **SCC:** No between group difference in VOR gain with vHIT
Jauregui-Renaud (2019) (53)	• 47 DM T2; 26%; 58 years old • NR; 8 years; 13 PN • 50 HC; 50%; 56 years old	**Utricle:** No between group difference in error of static SVV testing. Significantly more error in HC group vs. DM group in dynamic SVV during off-axis rotation. **hSCC:** Significantly lower VOR gain in DM group at.16 Hz, but not 1.28 Hz, vs. HC
Li (2019) (54)	• 51 DM T2; 47%; 56 years old; 24 kg/m^2^ • 8.5%; 11 years; 12 PN • 43 HC; 40%; 54 years old; 24 kg/m^2^ • 5.7%; NR	**hSCC:** 29 (57% of sample) of DM group with abnormal caloric tests (vs. 27% HC)
Moossavi (2021) (55)	• 15 DM T1; 60%; 28 years old • 8%; 10 years; NR • 16 HC; 19%; 26 years old	**Utricle:** Significantly longer latency with oVEMP testing in DM group. No difference in oVEMP inter-amplitudes. No difference in error with static SVV, but worse error with dynamic SVV (OKN) in the DM group. **Saccule:** Significantly lower inter-amplitude with cVEMP testing in DM group. No difference in latency. **SCC:** No difference in VOR gain with vHIT
Mahalingasivam (2023) (56)	• 52 DM T1; 50%; 59 years old, 26 kg/m^2^ • 8.2%; 28 y • 51 DM T2; 35%; 66 years old; 30 kg/m^2^ • 6.6%; 11 y • 11 HC; 54%; 59 years old; 25 kg/m^2^	**SCC:** No group differences VOR gain with vHIT. No subgroup differences in VOR gain with vHIT based on autonomic, large, or small fiber PN.
Zhang (2023) (57)	• 89 DM T2; 36%; 53 years old; ≈23 kg/m^2^ • 8.6%; 4 years; 29 w/o PN • 8.7%; 5 y: 26 symptomatic PN • 10.9%; 7 years; 34 asymptomatic PN • 42 HC; 45%; 52 years old	**Utricle and Saccule:** Significantly longer oVEMP and cVEMP latencies in the DM groups with PN vs. DM w/o PN or HC groups. No group differences in inter-amplitudes and a similar rate of absent VEMP responses between DM and HC groups.

BMI, body mass index; HbA1c, hemoglobin A1c; DM, diabetes mellitus; PN, peripheral neuropathy; T1, type 1; T2, type 2; NR, not reported; hSCC, horizontal semicircular canal; HC, healthy control; w/comp, with complications (included PN, ulcer, hemiparesis); MNSI, Michigan Neuropathy Screening Instrument score; OKN, optokinetic.

Sample characteristics as well as testing results organized by end-organ pathway are presented.

#### 4.1.1 DM type

We identified 10 articles describing people with Type 1 DM and 15 including people with Type 2 DM. Type 1 DM was the focus of cohort and case control studies until 2015 (8/9 studies); after which 12/13 studies included people with Type 2 DM (Table 4). Given this chronological distribution and more recent clinical implementation of cervical and ocular vestibular evoked myogenic potential (VEMP) and multi-canal head thrust tests, the horizontal semicircular canal (SCC) was the most frequently assessed vestibular end-organ in people with Type 1 DM. In these studies, some degree of abnormal caloric responses (low frequency) were consistently reported (5/5 studies), 1/3 reported vestibulo-ocular reflex (VOR) abnormalities with higher frequency SCC tests (35, 36, 39–42, 55, 56), and 2/2 reported abnormal saccule and/or utricle function (44, 55). In contrast, the otolith received more attention than the SCCs, and SCC assessments were across a range of frequencies (low, middle, high; Table 2) (17) in people with Type 2 DM. Specifically, 10/11 studies detected some form of otolith dysfunction (43, 45–53, 57), and 4/7 studies identified SCC dysfunction (40, 48, 49, 52–54, 56). While one study did not report DM type and another did not differentiate between types (37, 38), two studies compared classes of DM; demonstrating similarity in high frequency SCC testing (passive/active head thrust, video head impulse testing: vHIT) between people with Type 1 and 2 (40, 56).

Based on the current research, it appears there is a reasonable likelihood of detecting SCC or otolith dysfunction in people with Type 1 or 2 DM. While otolith dysfunction may be more likely than SCC dysfunction in people with Type 2 DM, SCC high frequency responses appear similar between DM types. Overall, differential end-organ effects between DM classifications are not apparent.

#### 4.1.2 Age, sex, and body mass index

Compared to sex and BMI, participant age was the most consistent reported factor in study designs (Table 4). Average DM participant age ranged from 16 to 66 years old, and often matched healthy control participant ages within studies. Consistent with younger Type 1 onset, and with the added benefit of controlling for the possible effect of age on vestibular function, studies including people with Type 1 DM were overwhelmingly younger than 50 years old. The youngest type 2 DM cohort age was 37, and most studies reported a mean group age of greater than 50; entering the age range when the VOR begins to decline regardless of DM (32). Sex was less frequently reported than age and not as frequently matched across groups. Female representation ranged from 8%–88%, but more often ranged between 30% and 60%. BMI was only reported in 6/23 reviewed studies (46, 49, 54, 56, 57). While the importance of age is implicitly recognized by authors who matched or controlled for age within designs, we are unaware of studies directly considering the possible interaction of age and DM with vestibular function. Additionally, we did not identify a study considering sex or BMI as a factor or covariate in analyses.

At present, the effect of age on study interpretation is somewhat mitigated, but it is unknown if an effect of sex or BMI underlies between study discrepancies regarding the characterization of abnormal vestibular function in people with DM.

#### 4.1.3 Blood glucose and DM duration

Blood glucose level was consistently reported as an inclusion criterion for DM group participants and disease duration was considered by many researchers as a way to either avoid or leverage the cumulative effect of hyperglycemia (Table 4). Focusing on the more contemporary clinical marker measuring blood glucose levels across the past three months, HbA1c, only 9/23 studies reported actual values in sample descriptions. Of the available data, all but three of the patient groups were above 8%; indicating a more severe level of disease that would be anticipated to increase the likelihood of detecting vestibular damage. To this point, 8/9 of these studies detected either otolith or SCC abnormalities in people with DM. Surprisingly, one study including people with Type 1 DM with 8.2% HbA1c, and the longest reported duration of reviewed studies (28 years), did not detect horizontal SCC dysfunction compared to people with Type 2 DM and a small subset of healthy controls (56). However, this study may not necessarily be an outlier, as a similar frequency of abnormal findings were observed in those across disease duration. Specifically, 6/7 cohorts with at least ten year and 5/7 with less than 10 year DM duration were reported to have some degree of otolith and/or SCC dysfunction. A trend toward earlier vs. later otolith or SCC onset was not evident (i.e., order effect). Studies employing correlation or factor level (i.e., high vs. lower HbA1c) analyses have attempted to address the ambiguity regarding the affect of HbA1c and DM duration. However, only 4/9 studies identified significant, small to medium effects, of these factors and VEMP latencies (2 studies) or caloric testing metrics (39, 45, 54, 57). Synthesizing this information suggests HbA1c may be a more robust predictor of vestibular dysfunction than DM duration, but inconsistency of predictions reduces confidence regarding this possibility.

Lack of consistent evidence connecting HbA1C or DM duration to vestibular function may be due to study design, the nature of DM, or their interaction. Design factors include sample heterogeneity and inadequate statistical power; the latter of which limits the ability to confidently consider multiple factors likely needed to predict tissue damage. Moreover, it is possible the timing of vestibular testing in relation to disease progression or HbA1c test influences relationships. Specifically, the degree of incremental or frequency of sporadic insults (e.g., hyperglycemic events) (39) to the inner ear and the response of the inner ear regarding vascular adaptations to metabolic stress and the possibility of spontaneous, yet incomplete, recovery are not the same across time in a person with DM or between people with DM. For example, different test results may be found in someone immediately after a series of hyperglycemic events or months of poor glucose control as compared to the same person after years of subsequent adequate glucose control. Somewhat fortunately, larger sample sizes with well-informed inclusion criteria and design (e.g., >7 years disease duration, standard time of HbA1c testing) may be the best approach to develop the profile of a patient who may need vestibular screening: mitigating the effect of more unique disease courses and allowing for the inclusion of multiple factors. To this point, in a promising study of 89 individuals with Type 2 DM, Zhang et al. (57) employed a Cox regression model that included HbA1c, additional blood markers (e.g., cholesterol), and severity of peripheral neuropathy to predict cVEMP latencies. Replication of this type of an approach may prove quite informative.

#### 4.1.4 Peripheral neuropathy

A number of studies have considered the presence of PN of the feet as a potential clinical surrogate marker of anticipated inner ear dysfunction (Table 4). The level of consideration has ranged from intentional exclusion, to simply reporting on the proportion within the sample, to designing studies to determine the effect of PN on vestibular test outcomes. Two studies detected otolith dysfunction, but a third did not detect SCC dysfunction, in people with DM without PN in comparison to controls (45, 46, 52). Across 11 study samples, and not necessarily controlled, the proportion of people with PN has ranged from approximately 30%–70%. Of these, six studies directly considered PN in comparison or correlational analyses. While otolith function in people with DMPN was worse than controls in 3/4 studies, discrimination of DMPN and DM without PN was only detected in 1/4 studies (43, 44, 50, 57). Two studies did not detect differences in high frequency SCC function as measured by VOR gain (vHIT) between healthy controls and people with DM, regardless of PN status (50, 56). Lastly, while Ward et al. (48) acknowledged the potential for insufficient statistical power, significant correlations between clinical scores of PN (Michigan Neuropathy Screening Instrument) and otolith and SCC function in people with DM were not observed.

Despite the consistent use of valid diagnostic tests of neuropathy (e.g., nerve conduction testing), limited presentation of data limits certainty regarding the clinical utility of considering PN related to inner ear function. However, we suggest stratifying people with DM based on severity of PN may elevate certainty. In illustration, VOR gain (vHIT) differences between people with Type 1 or 2 DMPN, verified by nerve conduction testing, and a small healthy control group, were not observed (56). Importantly, neither DMPN group registered mean vibration perception threshold test values worse than the known cut-off for loss of protective sensation, indicating the sample had a mild to moderate level of PN. In contrast, stratification of people with DM, DMPN with symptoms, and DMPN without symptoms (indicating more advanced PN), discriminated between groups and is a key predictor of cervical VEMP latencies (57). While it is possible PN affects vestibular end-organs differently, it is just as possible advanced PN (e.g., loss of protective sensation) is a surrogate clinical indicator of vestibular end-organ decline. Regardless, as it stands, otolith dysfunction can exist in the absence of PN, otolith dysfunction may be worse in people with advanced DMPN than in people with DM, and SCC dysfunction has not been detected in those with DMPN. Prudent next steps include replication of otolith assessments and a more comprehensive assessment of SCC function in people with DM and different levels of PN.

### 4.2 Where do we look for peripheral vestibular dysfunction?

Reviewing literature regarding the location of peripheral vestibular dysfunction in people with DM may provide clues to elevate efficiency of clinical testing. However, different study samples, designs, testing approaches, and the reality that only 4/23 studies considered each end-organ pathway within the same sample, will be reflected in our ability to make recommendations (48, 50, 52, 55). Nevertheless, studies are fairly balanced by end-organ as 10 studies assessed utricle function, 10 assessed saccule function, and 16 assessed SCC function (Table 4).

#### 4.2.1 Utricle

Six studies employed oVEMP testing with a median DM group sample size of 27.5 and age of 48.5 years (45, 48, 50, 52, 55, 57). Tone burst VEMPs were the most commonly employed stimuli, although intensity varied across studies. Of these, all but one small case-control study (*N* = 16) (52) found abnormal utricular responses in people with DM. Significantly delayed latency, or a greater number of abnormal latencies, were the most common finding in people with DM compared to controls, and in one study comparing DMPN to DM and controls (45, 48, 55, 57). Perhaps due to differences in approaches, two studies identified low amplitude responses in people with DM vs. controls (48, 50).

Four studies conducted tests within the subjective visual vertical (SVV) paradigm with a median DM group sample size of 47 and age of 57.5 years (46, 49, 53, 55). Three studies failed to detect differences in static SVV (46, 53, 55). While the fourth and largest (*N* = 152) study found a significantly larger DM group static error, the group mean was within the typical normative range of 0–2 degrees (18, 49). In contrast, all studies identified abnormalities with dynamic SVV testing. Suggesting visual dependence, two studies identified worse responses in DM group SVV with altered visual stimuli (tilted frame, moving background) (46, 55). And in suggestion of central compensation, Jauregui-Renaud et al. (49, 53) found worse bilateral SVV responses in people with DM during unilateral centrifugation as illustrated by less deviation from static values in comparison to control participants.

Nearly all studies detected abnormal utricular function. The greater frequency of abnormal oVEMP latencies vs. amplitudes points to nerve conduction deficits as opposed to signal dampening. Somewhat conversely, SVV findings point to a greater likelihood of both visual dependence and central compensation, suggesting that dampened signals have been reintegrated. Since oVEMP and SVV testing were conducted in the same cohort only once, further work is needed to clarify utricular pathway changes (55). However, slower conduction and compensation of utricular signals seem likely in people with DM.

#### 4.2.2 Saccule

The saccule was assessed with cVEMP testing across a median DM group sample size of 27.5 and age of 51 years. Here again tone burst VEMPs were the most commonly employed stimuli, but intensity was more consistent than in oVEMP tests across studies. Two studies did not find cVEMP abnormalities in people with DM, perhaps due to stimulus parameters or a small sample size (43, 52). However, 8/10 studies found group differences in either, but not both, amplitude or latency. Lower amplitude in people with DM vs. controls was observed in four studies, with an across study 0%–50% range of absent responses in the DM groups (47, 48, 50, 55). Significantly longer DM group latencies were observed in 3/10 studies; while another observed abnormally long latencies in 25% of their DM sample without dizziness (44, 45, 51, 57).

Based on the current research, most studies identified abnormal saccule pathway function in people with DM as measured by cVEMP testing. However, lower amplitude or longer latencies seem equally likely. Interestingly, lower amplitudes were found in cohorts with longer disease duration. Further, those with more advanced PN (and also higher blood glucose level) had longer latencies than those with less advanced PN or those with DM without PN (57). Perhaps, timing of testing with respect to otolith adaptations to metabolic stress explains between study discrepancies. Larger sample sizes may assist in clarifying expectations regarding cVEMP test results in people with DM.

#### 4.2.3 Semicircular canals

Eight studies employed caloric testing with a median DM group sample size of 30 and age of 26 years (35–39, 41, 42, 54). Authors of 6/8 studies reported abnormal caloric responses in people with DM, most often with a greater frequency than control subjects (35, 36, 39, 41, 42, 54). While slightly different methodology and test criteria were used, the range regarding the frequency of abnormal responses was 36%–60% in people with DM. Notably, the results of the lone study including people with Type 2 DM or older than 50 years of age were in line with the younger Type 1 cohort data of other studies (54).

Three studies employed rotational chair testing with a median DM group sample size of 64 and age of 60 years (40, 49, 53). Across two studies using rotational chair sinusoidal harmonic acceleration frequencies of 0.16 and 1.28 Hz, lower DM group VOR gain was observed in one cohort at 0.16 Hz (49, 53). In the third study, VOR gain at an unreported rotational frequency was not different between people with DM and controls; although phase differences were noted between groups of people with DM and controls (40).

Five studies used passive multi-canal high-frequency testing with a median DM group sample size of 25 and approximate age of 63 years (48, 50, 52, 55, 56). Group differences in VOR gain were not detected in the four studies employing vHIT testing between people with DM and controls. A small number of abnormal responses were noted in two studies (55, 56), while the other two studies reporting similar between-group VOR gain also reported no evidence of DM group covert or overt saccades (50, 52). However, utilizing a disappearing “E” paradigm, Ward et al. (48) demonstrated reduced dynamic visual acuity (DVA) in people with DM compared to controls. Horizontal and superior (anterior) canals were both different between groups, whereas the posterior canal was not. Combined, 70% of the DM cohort had at least one abnormal canal. Discrepancies between studies may be due to test or sampling approaches.

Overall, across low, middle, and high frequency SCC testing, people with DM had abnormal function in 9/16 studies. The most consistent case for horizontal SCC dysfunction was evident with low frequency caloric testing. Limited investigations point to the possibility of mid-range frequency horizontal SCC canal dysfunction, but the typical range of sinusoidal harmonic acceleration frequencies have yet to be employed. This is surprising since rotational chair testing is the standard for detecting bilateral vestibular loss (19); and bilateral loss is the theoretical expectation regarding the effects of chronic hyperglycemia. This limitation withstanding, behavioral VOR tests (e.g., DVA) requiring cortical/subcortical sensory integration, were convincingly abnormal in people with DM in one study (48). However, abnormalities with less complex assessments of high frequency VOR were not observed (vHIT). Together, findings suggest VOR is sufficient at frequencies needed for daily activities, but that multi-sensory integration of the VOR may be problematic. Relatedly, although clear in one study (56), greater transparency regarding how saccadic responses are defined may further understanding on if central compensation of SCC signals occurs in people with DM.

### 4.3 Why screen for vestibular dysfunction?

Visual, somatosensory, and vestibular systems are the primary sensory inputs for balance, and thus important factors to consider in fall prevention programs. Balance rehabilitation and fall prevention are particularly important in people with DM as falls are more frequent (25% vs. 18%) (59) and more likely to reoccur in comparison to people without DM; and falls in themselves propagate severe injury and elevate medical costs (8–10, 60–63). Moreover, the subclinical or overt compromise of visual and somatosensory systems (e.g., retinopathy, PN), along with impaired sensory integration, are well recognized major drivers of imbalance and falls in people with DM (58, 64, 65). Unfortunately, therapeutic approaches to reverse the effects of altered afferent information due to retinopathy and PN are unknown. In contrast, established exercises allow for the central reintegration of altered peripheral vestibular signals to allow for sufficient reflex responses (14). Therefore, if a connection between peripheral vestibular function and balance is identified in people with DM, vestibular exercises may prove to be a viable adjunct to current balance rehabilitation programs.

However, we are aware of only four studies including both peripheral vestibular diagnostic testing and balance assessments (40, 49, 52, 54). Nicholson et al. (40) found abnormal VOR phase during active or passive head rotations (horizontal SCC) and increased postural sway in people with DM vs. controls. In a small case-control study of a relatively young and early stage DM patient group, Omar et al. (52) observed a trend toward worse VEMP amplitudes and no group difference in VOR (vHIT) along with worse performance on clinical measures of balance (Timed up and Go test and Functional Gait Assessment). Li et al. (54) observed a greater frequency of abnormal horizontal SCC function (calorics) along with small but significant deficits in postural control in people with DM compared to controls of a similar age, sex, and BMI. In the largest case-control study, people with DM registered worse otolith (SVV) but not horizontal SCC (rotational chair) function along with worse postural control (49). However, postural control was not different between people with DM with (*n* = 26) and without (*n* = 75) a history of falling; and utricle function was not compared between these subgroups. In total, leveraging this literature to explain the possible relationship between vestibular function and balance in people with DM without dizziness is challenging due to differences in vestibular and balance metrics, and because direct analyses (e.g., correlations) were not conducted.

At present, evidence vestibular dysfunction is related to imbalance in people with DM is limited and circumstantial at best. As such, it is difficult to justify vestibular diagnostic testing in the absence of patient dizziness symptoms. While we acknowledge the potential need to consider a level of bilateral loss impacting function without symptoms of dizziness, and the likely summative effect of multi-sensory system compromise, further work is needed to clarify the potential role diagnostic vestibular testing has on the treatment of imbalance in people with DM.

## 5 Discussion

Our commentary has focused on which factors may increase the likelihood of vestibular dysfunction, where the dysfunction may be located, and to what extent said dysfunction may influence functional mobility in people with DM. We provided small summaries to this effect for each section and subsection thus far. Now, briefly synthesizing information across all sections allows us to offer recommendations for future research and current clinical practice.

Although we have illustrated the likelihood of detecting abnormal peripheral vestibular function in people with DM is relatively high, study findings are somewhat incongruent with anticipated DM pathophysiology regarding a progressively worse bilateral vestibular loss. Vestibular dysfunction was detected in 18/23 studies. However, qualitative review of the available data suggests unilateral changes are at least as common as bilateral changes in people with DM. This pattern may reflect reality, patient subsets, limitations of current diagnostic tests, or some combination. Regardless, because we only reviewed studies including people with DM without dizziness (or at least minimally so), and dysfunction can include utricle, saccule, and/or SCC pathways, compensation of both asymmetric and potentially symmetric dysfunction in any end-organ pathway seems evident. Further, based on promising but inconsistent results regarding glycemic control, and minimal evidence regarding DM duration, stronger evidence is needed to conclude vestibular dysfunction progressively worsens in people with DM. As such, vestibular dysfunction is present in people with DM with minimal to no symptoms (i.e., compensated), but not necessarily bilateral or progressive in nature.

The nature of vestibular insult aside, a major impetus of delineating vestibular dysfunction in people with DM is rooted in the possibility dysfunction may reduce balance and physical activity. As discussed (4.3), the connection is essentially unknown. However, inspection of study findings can provide preliminary clues as to how a connection may be present. Specifically, utricular dysfunction was more common than saccule or SCC dysfunction, and behavioral test abnormalities (SVV, DVA) were robust in people with DM. The frequency of utricular dysfunction is concerning given the emerging role this pathway has in patient recovery following vestibular insult (66, 67). And impairments on behavioral tests point to sensory integration difficulties. Combined, these test results raise the probability that vestibular dysfunction would manifest as imbalance in people with DM. Accordingly, incorporating such a test profile into research may serve to inform the future clinical care of people with DM.

### 5.1 Research recommendations

Based on the state-of-research, a number of strategies are recommended to move the field forward. Large samples within a longitudinal design, or cross-sectional stratified sample designs of people with differing severity of DM, may mitigate test timing concerns and reveal the sequencing and laterality of vestibular insult. Composite metrics of DM status, such as variability of HbA1c across time or average HbA1c normalized to disease duration, as well as levels of PN or unexplored measures of AGEs (31), may prove to be an effective way to explain and predict vestibular dysfunction toward informing test indications. Further, assessment of all three end-organ pathways within the above recommended designs remains necessary until the requisite clarity is achieved to optimize clinical testing paradigms. Low to mid-range SCC and otolith pathway tests along with behavioral tests (SVV, DVA) may best position us to link dysfunction to imbalance in people with DM. To this point, conduction of multiple regression analyses are needed to evaluate if vestibular dysfunction accounts for imbalance and functional mobility. Ultimately, we suspect a multisensory assessment of somatosensation/proprioception, vision, and vestibular function will be needed to establish the unique contribution of the vestibular system to balance in people with DM.

### 5.2 Clinical recommendations

Despite the uncertainty regarding the contribution of vestibular dysfunction to imbalance, there are a number of reasons to consider the clinical evaluation of vestibular function in people with DM and imbalance with or without dizziness. These reasons include, (1) the high likelihood of vestibular dysfunction, (2) uncertainty regarding possible morphological adaptations to metabolic stress, (3) the degree of anticipated compensation, and (4) importance of multi-sensory integration for balance ability. Therefore, in addition to a through history to determine provoking factors/activities of imbalance, we recommend the incorporation of bedside exam tests prior to instrumented diagnostic testing. Simple oculomotor tests (i.e., pursuit, gaze, VOR cancellation, optokinetic response), DVA testing, and static and dynamic balance tests that require an integrated vestibular response are reasonable to include during patient evaluations. We expect such an approach will allow clinicians to add vestibular exercises and based movements, as indicated, to current approaches aimed at improving balance in people with DM (68, 69). Finally, in cases where individuals do not respond to this type of an approach, use of vestibular diagnostic testing may be of some benefit.

### 5.3 Limitations

A full review of DM pathophysiology was beyond the scope of this review. We acknowledge study findings of central vestibular dysfunction in people with DM and encourage clinicians to consider this possibility during patient care (35, 36, 39–42, 70). Some studies on peripheral vestibular function in people with DM were excluded due to the inclusion of people with unspecified vertigo or dizziness, either via discovery or as designed (71–75). Additionally, we recognize DM increases the likelihood of benign paroxysmal positional vertigo and appears to worsen the prognosis of Meniere's disease (76–78). These studies indicate not all vestibular dysfunction in people with DM is peripheral, asymptomatic, or compensated. Lastly, while study methodology and quality were considered within this review, detailed commentary on methodology or criteria-based quality rankings were not conducted.

### 5.4 Conclusion

We offer mitigated conclusions regarding when, where, and why we should look for vestibular dysfunction in people with DM. It appears peripheral vestibular dysfunction is likely in people with both types of DM. It also appears greater HbA1c and severity of peripheral neuropathy increases this likelihood. Both otolith end-organs and the SCCs are candidates for dysfunction. However, it is quite uncertain if anticipated vestibular dysfunction manifests as imbalance in people with DM.
